# *How different are parents and educators*? A comparative study of interactive differences between parents and educators in a collaborative adult-child activity

**DOI:** 10.1371/journal.pone.0205991

**Published:** 2018-11-14

**Authors:** Marina Fuertes, Otília Sousa, Marta Łockiewicz, Clarisse Nunes, Dalila Lino

**Affiliations:** 1 Instituto Politécnico de Lisboa, Escola Superior de Educação de Lisboa, Lisboa, Portugal; 2 University of Porto, Centro de Psicologia, Porto Portugal; 3 Universidade de Lisboa, Instituto de Educação, UIDEF, Lisboa, Portugal; 4 University of Gdańsk, Institute of Psychology, Gdańsk, Poland; 5 Universidade do Minho, Centro de Investigação em Estudos da Criança, Braga, Portugal; TNO, NETHERLANDS

## Abstract

Involving children in collaborative tasks supports their cognitive, motor and social development. This study, performed in Portugal, aims to describe and compare early childhood educators and parents regarding their collaborative and interactive behavior when working with children. For that purpose, 55 educators (of both genders) with a child from their class and 45 parents (of both genders) with their children, participated in an everyday-like quasi-experimental situation for 20 minutes. The participants were invited to build an object of their choice, using a range of available materials and tools. The children included 47 boys and 48 girls, between 3 and 5 year-old. In comparison with the parents, the educators encouraged the children more to explore and find their own solutions. Conversely, the parents helped their children by offering demonstrations and directions. When the educators and the parents were grouped by gender (“men” versus “women”), different opportunities were offered to boys and girls by male and female adults. Our study suggests that educators and parents serve as diverse, but complementary educational role models and provide different learning opportunities.

## Introduction

Children live in and are influenced by several contexts, while affecting them. Each context constitutes an environment with its own culture, in which many people play different roles in children´s lives. Children learn to coexist in these contexts, organizing “internal representation models” of their agency in the world and of their relationships [1].

Typically, the first life context is family. Parents introduce their children to daily social and learning opportunities which allows the development of emergent competencies [2]. Another key context is Early Childhood Education (ECE), a pedagogical preschool setting that aims to promote learning opportunities in children’s daily routines and interactions [3, 4]. Given that both educators and parents contribute to a child´s development and learning, it is important to examine and compare their contributions. Such studies, however, have rarely been conducted. On the other hand, collaborative tasks offer children important social, cognitive and motor experiences [5]. Therefore, we aimed to examine parents´ and educators´ interactions with children during such tasks. We intended to investigate, how adults engage, motivate and involve children in collaborative tasks? Are there any differences in parents’ and educators’ behavior?

### Educators and parents

Lilian Katz [6] described the significance and complementarity of parents’ and educators’ roles in child development and learning. The author established seven categories differentiating parental and educator’s roles: *function*, *intensity of affect*, *attachment*, *rationality*, *spontaneity*, *partiality* and *responsibility*. Parents are, in most cases, attachment figures. Thus, working without a script, the parents are spontaneous, biased by their love, and responsible for the child’s safety and well-being [6]. Educators, however, perform a professional role guided by pedagogic intentionality [7]. Thus, they are more focused on a group-oriented practice, rather than on an individual, affective relationship with each child. According to evolutionary psychology, parental behavior is motivated by evolutionary interests [8–10]. Therefore, parents are biologically committed to prepare their children to pursue personal goals, while educators prepare their pupils to become socially integrated and valuable for society. Of course, cooperation with others is fundamental, either when one acts in their own best interest or when one is socially motivated [11]. In fact, both contributions influence each other and are important for a child’s development, including school education, active citizenship, social interactions, establishing personal goals and cooperation with others.

### Interactive behaviors, cooperative tasks and active learning

Previous research indicated that the quality of educator-child interactions can exert a positive impact on children’s learning and development [12]. However, only meaningful interactions, in which adults create an interpersonal, supportive environment that includes: active listening, reciprocity, mutual respect and participation, can actually contribute to children’s social, emotional, cognitive and motor development [13, 14].

In Portugal, a study inspired by Bertram and Pascal’s studies [15, 16] and the Laevers’ scale [17] described educator-child interaction regarding *Sensitivity* (adults’ attention regarding a child’s feelings and well-being), *Stimulation* (contents and adult actions in the learning process), and *Autonomy* (opportunities to explore, make judgments, choose activities and express ideas). Results indicated that more experienced [18–20] and following a social constructivist approach [21, 22] educators are more sensitive to children’s needs, and more likely to promote their autonomy, as compared with less experienced and more traditionally oriented ones. In constructivist-oriented teaching, educators promote collaborative interactions among children and involve children in collaborative tasks [23].

The significance of cooperative tasks and interactions for child development and learning is well described [5]. Cooperative tasks involve children in achieving common goals through working with others. To that end, children need to be involved in setting goals, planning, and making decisions while manipulating materials and tools [24, 25]. Cooperative tasks improve planning and problem-solving skills [25–29]. Moreover, children who perform better in cooperative tasks were more popular and socially accepted in their peer group [5]. Overall, these activities offer important opportunities to children, as they take proactive part in their own learning process and work as an equal partner with adults. Even though parents and educators involve children in collaborative tasks on a daily basis, according to our best knowledge, no other study compared parents and educators in working with children in these tasks.

### Children’s gender and education

Implicit and explicit parental and educators’ behaviors and communication may guide children’s socially constructed gender roles, behaviors, activities and attributes [30, 31].

Previous research indicated differences in mothers’ and fathers’ interactive behavior and play, related with children’s gender. Mothers are described as more affective and more likely to use negotiation in conflict solving situations than fathers are [32, 33]. Fathers tend to be less attentive than mothers, but challenge their children more to explore new situations and to compete [34, 35].

Similarly, female and male educators differ, as male educators tend to propose more motor and physically challenging activities than female educators do [36–38]. One study found that products made by a child displayed more autonomy, creativity and originality, when collaborated on with a male, rather than a female, educator [39].

Furthermore, fathers, mothers, male and female educators adapt their behavior when interacting with boys or girls to the child’s gender. For example, fathers engage in more physical and active play [40, 41] and are more unpredictable [42] with their sons than with daughters [43, 44]. In turn, mothers promote more symbolic play [45, 46] and are more emotionally available with their daughters [47].

Inspired by the differences in male and female behavior with children, a German team (*Tandem Team*) studied male educators and female educators in a collaborative situation named the *Tandem situation* [48]. Their findings indicate that female educators tend to use more fantasy with girls and to communicate in a more objective and concrete way with boys. To learn more about educators’ and parents’ differences and gender contribution to children learning and active participation in collaborative interactions, we added parents as participants to our research.

#### Present study

Although previous studies indicated differences in parents’ and educators’ behavior, none examined parents and educators in cooperative tasks. This study aimed to describe and compare educators’ versus parents’ interactive behavior, regarding *Empathy*, *Challenge*, *Attention and reciprocity*, *Cooperation*, *and Communication quality*. Additionally, we aimed to investigate the authorship of the product (mainly produced by the child, by the adult or made by both in cooperation). To study gender effects, the participation of the child with the female and the male adults was compared. Moreover, we analyzed adult’s behavior when paired with boys and girls. Finally, we aimed to study the association between the demographic variables (e.g., a child’s age, the number of siblings, an adult’s age, adult’s years of education) and educators’/parents’ interactive behavior.

## Methods

### Participants

This Portuguese research included two groups: *Parents* (fathers and mothers) and *Educators (males and females)*. In the *Parents group*, 22 fathers and 23 mothers participated and in the *Educators group*, 10 male educators and 40 female educators participated. The scarcity of male educators in the study is due to the difficulty of finding men working as early childhood educators (a mandatory requisite to participate in the study). Each educator was observed with a randomly selected child from their class and each parent with their own child. Each participant participated only once.

Educators and children had known each other for at least 6 months prior to the study.

Only children without developmental problems or delays were included in the study.

Table 1 presents the demographic information separately for each group. The data indicate that the groups were similar, with the exception of the number of siblings.

**Table 1:** Demographics.

Items	Educators		Parents	
	*M (min*.*-max*.*)*	*SD*	*M (min*.*-max*.*)*	*SD*
Child age (years)	3.56 (3–5)	.45	4.29 (3–5)	.73
N.° of siblings	.41 (0–2)	.53	.80 (0–1)	.45
Adult age (years)	34.81 (25–45)	5.16	36.71(25–45)	4.44
Years of education	14.71(9–20)	3.23	15.75 (15–19)	.67

Adult participants (Parents and Educators) were from middle class households and only two parents were unemployed. Most participants were Portuguese, except for a parent from Eastern Europe and 2 parents from Africa (living in Portugal for more than 10 years). The educators had worked between 5 and 10 years and all of them had a bachelor’s or a master’s degree in Preschool Education.

### Procedure

During a first meeting, the aims and procedure of the study were explained to the parents and educators and their questions were answered by the researchers. Subsequently, an explanatory brochure was delivered to the participants. When adults had expressed informed consent for their own and their children’s (in the case of parents) participation, the children were contacted, and the aims of study where explained to them as well.

In the next meeting, following the APA standards, the educators, the parents and the children agreed to participate, all in a written form (a drawing in the case of the children). Before starting the Tandem observation, demographic data were collected in a brief questionnaire answered by parents and educators.

Adults’ and children’ decision to withdraw from the study was respected throughout the research, which was presented as a play task.

All ethics proceedings were approved and budgeted by Instituto Politécnico de Lisboa Research Bureau (IDI&CA 2016).

Each dyad (adult-child) was videotaped independently in an everyday-like quasi-experimental interaction (without the presence of the investigator) in a free activity of joint construction with pre-determined materials, following the original Tandem study protocol [49]. The only instruction given to all participants was to make a product (e.g., an object, a toy, a doll) of their choice with materials and tools available, within a limited time of 20 minutes. Materials and tools amount, exposition, space and chair positions were controlled in each observation.

The participants had access to two suitcases, one containing several materials (wooden planks, colorful paper, fishing line, ribbons, self-adhesive eyes, colorful beads, sticks, corks, corrugated paper, felt, pipe cleaners, wire, egg box, polystyrene balls, toilet paper tubes, wool, straws, and metal rings), and the other containing tools (hot adhesive pistol, plier, hammer, glue, and scissors). Identical materials and tools were given to each dyad. Moreover, a chronometer was available to help the participants monitor the task time. The observations were collected in the child care centers or kindergartens attended by the children. The research assistants made sure that no distraction or interruptions occurred during the observation.

### Scoring

To study the quality of adult interactive behavior, we used a *Tandem Adult Interactive Scale* [49], translated and adapted to Portuguese by Rita Brandes. The scale consists of 18 items representing six dimensions: Empathy; Challenge; Attention and reciprocity; Cooperation; and Communication quality (cf. Table 2). Each item is scored from 1 to 5 points on a Likert scale (1 point corresponds to “strongly disagree”; 3 points to “partly agree”; and 5 points to “strongly agree”). After watching the video, two female coders and two male coders (to ensure gender balance of the scoring) with educational training scored each item individually. Next, item by item, the coders presented their scores and discussed their score item by item. The final scoring was obtained by agreement of the four coders.

**Table 2:** Dimensions and items of the *Tandem Adult Interactive Scale*.

Dimension	Corresponding items in each dimension
**Empathy**	1.1 The adults react to the expressions and emotions of the child appropriately & promptly
	1.3 The adult supports the child appropriately (unrequested interference or rules).
	1.4 The adult gives positive and appreciative feedback.
**Challenge**	1.2 The adult encourages the child to explore and analyze new problems.
	2.3 The adult asks questions which stimulate reflection/thinking
	2.4 The adult introduces new concepts/terms
	3.5 The child loses interest in the activity and reveals signs of boredom.
	3.6a The adult organizes the activity as an achievement-oriented situation
	3.6bThe adult competes with the child during the game
**Attention and reciprocity**	2.1 The adult adopts suggestions and/or initiatives of the child
	2.2 The adult waits patiently for decisions of the child.
	2.8 The adult physically faces the child and seeks eye contact.
**Cooperation**	3.1 The adult observes the child and only participates verbally.
	3.2 The adult acts himself/herself and lets the child observe.
	3.3 The adult and the child pursue different sub-projects in parallel activity with only partial cooperation
	3.4 Both work together in an object, with a continuous conciliation of interests.
**Communication quality**	2.5 The adult expresses themselves, mainly, in an objective-concrete, and functional way about the activity or adopts it, when these comes from the child d.
	2.6 The adult accompanies the activity with associative fantasies and narratives or adopts them, when these comes from the child
	2.7 The adult thematises the relationship or personal aspects (attributes, experiences, feelings) or adopts them, when these comes from the child

### Data analysis

Data were computed using the SPSS 22. The descriptive statistics were used to calculate means and respective standard deviations. The *t-student test* was used to test for mean differences between the *groups* (Parents vs Educators; Males vs Females). Pearson correlations were used to describe the association of adults’ behavior and the demographic variables. The level of significance was assumed as .05 and the distribution of variables was tested to choose between parametric and non-parametric statistics.

## Results

Following our study aims, we tested: i) differences in the authorship of the product in dyads with parents and in dyads with educators; ii) the quality of the interactive behavior in dyads with parents and with educators; iii) gender differences in adults’ behavior, and iv) the association between adults’ behavior and demographics.

### Authorship of the product in the parent-child and educator-child dyads

We found that in educator-child dyads, the children largely either created products on their own (only with some support provided by an adult) or in collaboration with Educators, whereas in parent-child dyads, the children mostly observed while their parents created products (cf. Table 3).

**Table 3:** Distribution of children participation with their parents and with their educators.

Children participation with	Made by			Total
	*Child*	*Both*	*Adult*	
Educators	21	17	12	50
Parents	2	18	25	45
Total	23	35	37	95

### Relationship between the adult role (parent versus educator) and the quality of interactive behavior

The *Tandem Adult Interactive Scale* [49] was used to assess Parents’ and Educators’ interactive behavior. According to Table 4, there were significant differences in parents’ and educators’ behavior in most items (11 of 18).

**Table 4:** Descriptive statistic and results of group differences test (dyads with educators versus dyads with parents) for adult interactive behaviors.

Behavioral Categories	Interactive Behaviors	Educators-child		Parents-child		t
		*M*	*SD*	*M*	*SD*	
**Empathy**	The adult physically faces the child and seeks eye contact	3.98	1.19	3.11	1.21	3.671***
**Challenge**	The adult encourages the child to explore and analyze new problems.	3.38	1.21	2.51	1.14	3.944***
	The adult asks questions which stimulate reflection/thinking	3.20	1.10	2.51	.99	3.121**
	The adult introduces new concepts/terms	3.2	1.1	2.51	.99	3.526***
	The adult organizes the activity as an achievement-oriented situation	1.24	.57	1.82	1.17	-2.834**
	The adult competes with the child during the game	1.60	1.93	2.36	1.31	-2.963**
**Attention and reciprocity**	The adult waits patiently for decisions of the child	3.73	1.25	3.00	1.26	2.770**
	The adult adopts the child’s suggestions/ initiatives of the child	4.04	1.19	3.27	1.96	3.099**
	The child loses interest in the activity and reveals signs of boredom.	1.51	.89	2.09	1.5	-2.669*
**Cooperation**	The adult observes the child and only participates verbally	2.93	1.25	1.13	.34	6.316***
	The adult acts himself/herself and lets the child observe	2.13	1.16	2.89	1.4	-2.786*
	The adult and the child pursue different sub-projects in parallel activity with only partial cooperation	1.53	1.14	2.07	1.10	-2.275*
	Both work together in an object, with a continuous conciliation of interests	4.02	1.34	2.96	1.24	3.821***
**Communication quality**	The adult thematises the relationship or personal aspects (attributes, experiences, feelings) or adopts them, when these comes from the child	2.84	.18	2.18	.16	3.741***

*p≤.05;

**p < .01**

***p < .001

Those differences are summarized in Table 5.

**Table 5:** Summary of interactive behavior differences in educators and parents.

Behavioral Categories	Educators tend to	Parents tend to
**Empathy**	Better eye contact and paying attention	
**Ability to challenge the child**	The adult encourages the child to explore and analyze new problems	Organize the task as a competition situation
	Ask questions that stimulate reflection /thinking	
	The adult introduces new concepts/terms	
**Attention and reciprocity**	Adopt the child’s suggestions/ initiatives.	
	Wait patiently for decisions of the child	
**Cooperation**	Observe the child and only participate verbally	Act himself/herself and let the child observe
	Work together with the child in an object, with a continuous conciliation of interests	To pursue different sub-projects in parallel activity with only partial cooperation
**Communication quality**	Thematises the relationship or personal aspects (attributes, experiences, feelings) or adopts them, when these comes from the child	

### Relation between gender and the quality of an adult’s interactive behavior

No significant differences were found regarding Parents’ and Educators’ interactive behavior with boys or girls. However, when we analyzed the adults’ behavior by gender, grouping male participants (i.e. male educators and fathers) *vs* female participants (i.e. female educators and mothers), both groups acted differently with children (cf. Table 6). Men, as compared with women, tended to be more competitive, leading the activity and promoting more parallel projects. In turn, women more often maintained eye contact, and allowed the children to participate and to engage in collaborative work.

**Table 6:** Descriptive statistic and results of group differences test according to gender (women *vs* men) for adult interactive behaviors.

Behavioral Categories	Adult Interactive Behaviors	Women-child		Men-child		*t*
		*M*	*SD*	*M*	*SD*	
**Attention and reciprocity**	The adult physically faces the child and seeks eye contact	3.98	1.18	3.00	1.09	4.020**
**Cooperation**	The adult observes the child and only participates verbally	2.08	1.23	1.24	.43	4.018***
	The adult acts himself/herself and lets the child observe he adult acts and lets the child observe	2.17	1.22	2.97	1.37	-2.928**
	The adult and the child pursue different sub-projects in parallel activity with only partial cooperation	1.48	1.15	2.24	1.00	-3.261**
	Both work together in an object, with a continuous conciliation of interests	3.90	1.35	2.92	1.19	3.580**
**Communication quality**	The adult organizes the activity as an achievement-oriented situation	1.27	.66	1.89	1.18	-3.200**
	The adult competes with the child during the game	1.59	1.01	2.50	1.33	-3.687***

*p < .01;

**p < .005;

***p < .001

Interestingly, our results indicated that male and female participants acted differently with boys and girls. Often, the girls were given the opportunity to work in partnership with men. Conversely, the boys, while interacting with men, were relegated to an observer role, while the adult managed the activity (cf. Table 7). Such a tendency was completely reversed when the boys and the girls worked with women (cf. Table 8).

**Table 7:** Distribution of boys and girls participation with men.

Children Sex	Made by			Total
	*Child*	*Both*	*Adult*	
Male	1	8	14	23
Female	1	11	9	20
Total	2	19	22	43

**Table 8:** Distribution of boys and girls participation with women.

Children Sex	Made by			Total
	*Child*	*Both*	*Adult*	
Male	12	6	6	27
Female	6	10	9	34
Total	21	16	15	47

### Relation between demographic variables and interactive quality of a dyad

Out of several demographic variables analyzed (e.g., an educator’s number of years of professional experience, children’s age, number of siblings), only an adult’s age correlated with their interactive behavior (cf. Table 9).

**Table 9:** Pearson correlations between adult interactive behavior and the adult age (parents and educators).

Behavioral Categories	Corresponding items in each dimension	*R*
**Empathy**	- The adults react to the expressions and emotions of the child appropriately & promptly	-.253*
	- The adult supports the child appropriately (unrequested interference or rules).	-.254*
	- The adult gives positive and appreciative feedback	-.302*
**Challenge**	- The adult encourages the child to explore and analyze new problems	-.245*
	- The child loses interest in the activity and reveals signs of boredom	.207*
	. The adult competes with the child during the game	.265*
**Attention and reciprocity**	- The adult waits patiently for decisions of the child.	-.215*
**Cooperation**	The adult adopts suggestions and/or initiatives of the child	-.271*
	- The adult observes the child and only participates verbally.	-.246*
	- Both work together in an object, with a continuous conciliation of interests	-.311*

*p < .01;

## Discussion

The present study aimed to describe and compare the interactive behavior of 50 Educators and 45 Parents when working with children in preschool age, during an everyday-like cooperative construction activity. In the present discussion, we complement our quantitative results with qualitative examples and descriptions to better illustrate and interpret our results.

### Educators and parents interactive behavior

Our study indicates clear differences in Parents’ and Educators’ interactive behavior. Indeed, children are more likely to be active participants and main authors of product when paired with Educators, rather than with Parents. Educators tended to let the child decide what product would be built, and which materials/approach would be used to complete it. Furthermore, during these interactions, Educators used supportive and encouraging language to involve and encourage children, as the following example demonstrates:

**Educator (E)**: *And now let's start playing*! *With what do you want to play*? *Do you want to construct something or do you want to play*?

**Child (C)**: *I want to do something*.

**E**: *And what do you feel like doing*?

Educator is waiting patiently for the C’s answer.

**C**: *Hum … a necklace*.

**E**: *A necklace*? (Big smile) *Good*, *I think it's a good idea*. *And with what are we going to create the necklace*?

**C**: *With this (Picking up a box)*.

According to our observations, the parents and the educators used different educational models: the educators used a collaborative-oriented model of interaction, while the parents preferred a verbal instruction (i.e. giving suggestions) and imitation model (i.e. making the product themselves and letting the child observe). For instance, some parents decided to make the product requesting materials and suggestions from the child. In the next example, we present an interaction where a father made most decisions and set an example during the activity.

**F**: No need to! (the father tries to stick the eye, but without success. The child observes)

**F**: Let’s put some glue (he takes the glue from the child’s hands and glues the eye).

**F**: What about the doll’s nose?

**C**: The doll’s nose is round.

**F**: No, it is oval (the father draws it and the child leaves the working table, abandoning the activity).

The final product of this dyad is a very conventional doll with a very adult-influenced design.

According to Tomasello, Kruger and Ratner [29] cultural learning manifests in three forms: imitative learning, instructed learning and collaborative learning. It seems that with parents, learning relies on modelling and explanation [13, 50, 51]. Likely, they adopted this teaching style from their own parents. Yet, if only this transmissive practice is experienced, children tend to become less interested, engaged, and involved, as our results demonstrated. Indeed, our results indicate that children paired with parents lost interest during the activity (e.g., some left the table altogether or started a narrative not related with the task). In pre-school age, initiative is a major process and driving force of development [52, 53]. Thus, those children whose initiative is supported, are active, persistently pursue their goals, know how to cooperate with others and how to lead, and develop a basis for taking up later challenges set by others, e.g. in further education (e.g. [54]).

We hypothesize that professional training and reflective thinking about their teaching practices provided the Educators with several useful models which promote children’s participation [55]. For example, the social constructivism model highlights that learning is an active process, where learners actively collaborate with others in order to build their own knowledge [56, 57].

### Interactions within a specified period of time

In this study, interactions were not observed during daily events (at home or school). Thus, we cannot be sure if Portuguese preschool children have an opportunity to learn and play with their parents and with their educators for 20 minutes in a continuous, collaborative and dyadic activity. The educator-child ratio in preschool education in Portugal (1 to 14, on average) hardly allows these opportunities to happen with regularity. In our research [20, 58, 59], many parents stated that this was their first experience in working collaboratively with their children. Yet, our findings suggest that, given an opportunity, Educators and Parents tend to engage in positive and reciprocal interactions with children. We wonder if the quality of these interactions can increase over time and with experience. Possibly, within school-family co-operation, Educators could encourage parents to engage in collaborative tasks with their children at home, to both promote the development of cognitive and social skills of the children and to increase parent-child quality time.

### Gender opportunities

Contributing to gender research, our findings indicate differences in male (fathers and male educators’) and female (mothers and female educators’) participants interactive behavior with boys and girls.

Interestingly, the girls were more often offered the role of a partner and an opportunity for a cooperative work, while boys were either leaders or followed an adult’s leadership. In fact, adults often rejected boys’ ideas and suggestions. In addition, men, in comparison with women, were more competitive, performing the activity individually, letting the child observe, or opting for parallel projects. Some male educators and fathers set competitions to see what worked faster and, thus, better. Many children accepted the challenge in a playful manner. Conversely, women maintained eye contact, permitted the child to lead the activity, or worked in collaboration with the child. A body of research indicates differences in girls and boys behavior [60–62]. We wonder how many of these differences were shaped by learning and repeated, interactive and social experiences offered to them in the first years of their lives, like the ones that we observed in our study, especially as these gender-based influences were found to be similar in two important environmental contexts: family and pre-school.

Undeniably, we found multiple social and learning opportunities offered to the children. If the boys had fewer opportunities to actively participate in task performance when working with fathers and male educators, they gained that opportunity with mothers and female educators. Possibly, the boys learnt their place in the “male” hierarchy”, including waiting for their turn, observing not acting, and self-regulating their behavior. In mother-boy and female educator-boy dyad, the boys could be more autonomous and act as leaders. Future, longitudinal research may examine the influence of these experiences on children’s later social development, as well educational and careers choices.

### Developing a model

In the Introduction section, we presented Lilian Katz’s model [6], to explain differences in Educators’ and Parents´ behavior. In this model, parents are described as affective figures, spontaneous in their teaching and invested in their child’s interests. Conversely, educators, following their pedagogic intentionality and professional role, are more focused on group-oriented practices [7, 63]. Findings from our study corroborate this theoretical proposal: (i) the Educators preferred a group-oriented approach (stimulating collaborative practices) while Parents tended to promote self-interested behaviors (stimulating competition); (ii) throughout the task, the Parents’ behavior seemed more spontaneous, favoring imitative actions, while the Educators challenged the children to think about their decisions and ideas *(e*.*g*., *asked more questions that stimulated reflection/thinking* and *introduced the child to new concepts*); but (iii) Parents were more attentive and positive in responding to the children’s emotions.

Another corroboration of this model is present in a study comparing Female Educators paired with children from their classes, Female Educators/Mothers paired with their own children (sons or daughters) and Mother (with other jobs rather than Educators) in the same Tandem experience [64]. Surprisingly, according to that study, Educators with their own children (sons or daughters) observe less, participate more and relegate the child to the observer role (“*mother can do this for you*”; “*mother can help to improve your drawing*”). That study suggests that female Educators, when working in tandem with their own children, act mostly as Parents, and are less oriented by pedagogical approach.

In sum, children learn through experience and our findings bring new information to the debate about how to promote their participation and learning opportunities in cooperative activities. It is our hope that our findings contribute to discussion about training of ECE educators and the need to reinforce reflective practices in gender education and collaborative practices with families.

### Contributions for educators training

Our results indicate that educators, recognizing children’s competence and agency, give them opportunities to act autonomously and to make decisions. Moreover, educators used questions to enhance children’s reflection skills and to stimulate creativity. This style of educator-child interaction stems from a pedagogical approach that promotes children’s participation in their own learning and development through the processes of collaboration and cooperation with adults, in this case with an educator.

Nonetheless, in our study the average quality of the educator’s interactive behavior in most items of the *Tandem Scale* ranged from *moderate* to *good quality*, not reaching the highest level, *very good quality*. These data indicate a need for an analysis of the ECE teaching programs, to examine how adult-child interaction is integrated throughout the curriculum and how college/university students are trained to use an interaction approach that promotes collaboration between children and educators, thus promoting the co-construction of knowledge [13, 63].

Finally, in our study gender differences emerged when men or women (educators and parents) interacted with boys or girls. These findings point out the need for reflection about gender in ECE practices and curriculums in order to offer equal challenges and opportunities to girls and boys [38].

## Limitations and future directions

Although our findings contribute to a growing knowledge about parents’ and educators’ interactive behaviors in different cultures, our findings cannot be generalized to all populations. Moreover, the use of an everyday-like quasi-experimental situation is sensitive to the influence of a *record effect* and participants’ behavior, and does not involve an observation in daily contexts. Thus, our results can only be interpreted considering these observational conditions. The studies about the daily practice of the ECE Portuguese educators and the studies about Portuguese families rarely identify situations where children engage in 20-minute tasks with one adult [64]. Therefore, *Tandem situation* created a good opportunity to make such observations.

Another limitation can be identified in the groups. The distribution of male and female educators was extremely asymmetrical (40 female and 5 male educators). However, such asymmetry represents the ECE ratio in Portuguese population [65].

As future directions, our lab is dedicated to describe children behavior in these interactions with educators and parents of both genders.
